# Simplifying understory complexity in oil palm plantations is associated with a reduction in the density of a cleptoparasitic spider, *Argyrodes miniaceus* (Araneae: Theridiidae), in host (Araneae: Nephilinae) webs

**DOI:** 10.1002/ece3.3772

**Published:** 2018-01-03

**Authors:** Dakota M. Spear, William A. Foster, Andreas Dwi Advento, Mohammad Naim, Jean‐Pierre Caliman, Sarah H. Luke, Jake L. Snaddon, Sudharto Ps, Edgar C. Turner

**Affiliations:** ^1^ Department of Zoology University of Cambridge Cambridge UK; ^2^ PT SMART Tbk SMART Research Institute Pekan Baru Indonesia; ^3^ Durrell Institute of Conservation and Ecology (DICE) School of Anthropology and Conservation University of Kent Canterbury UK; ^4^ Centre for Biological Sciences University of Southampton Southampton UK

**Keywords:** agricultural management, habitat complexity, host–parasite relationships, population density, trophic interactions

## Abstract

Expansion of oil palm agriculture is currently one of the main drivers of habitat modification in Southeast Asia. Habitat modification can have significant effects on biodiversity, ecosystem function, and interactions between species by altering species abundances or the available resources in an ecosystem. Increasing complexity within modified habitats has the potential to maintain biodiversity and preserve species interactions. We investigated trophic interactions between *Argyrodes miniaceus,* a cleptoparasitic spider, and its *Nephila spp*. spider hosts in mature oil palm plantations in Sumatra, Indonesia. *A. miniaceus* co‐occupy the webs of *Nephila spp*. females and survive by stealing prey items caught in the web. We examined the effects of experimentally manipulated understory vegetation complexity on the density and abundance of *A. miniaceus* in *Nephila spp*. webs. Experimental understory treatments included enhanced complexity, standard complexity, and reduced complexity understory vegetation, which had been established as part of the ongoing Biodiversity and Ecosystem Function in Tropical Agriculture (BEFTA) Project. *A. miniaceus* density ranged from 14.4 to 31.4 spiders per square meter of web, with significantly lower densities found in reduced vegetation complexity treatments compared with both enhanced and standard treatment plots. *A. miniaceus* abundance per plot was also significantly lower in reduced complexity than in standard and enhanced complexity plots. *Synthesis and applications*: Maintenance of understory vegetation complexity contributes to the preservation of spider host–cleptoparasite relationships in oil palm plantations. Understory structural complexity in these simplified agroecosystems therefore helps to support abundant spider populations, a functionally important taxon in agricultural landscapes. In addition, management for more structurally complex agricultural habitats can support more complex trophic interactions in tropical agroecosystems.

## 1 INTRODUCTION

Tropical habitats are experiencing rapid change as the rate of agricultural expansion in the tropics increases (Foley et al., 2005; Gibbs et al., 2010; Hansen et al., 2008; Tylianakis, Didham, Bascompte, & Wardle, 2008; Tylianakis, Tscharntke, & Lewis, 2007). Oil palm is a leading tropical crop and has been responsible for the conversion of more than 10 million hectares of tropical forest over the past two decades (FAO 2010; Gibbs et al., 2010; Wilcove & Koh, 2010). Forest conversion causes severe variation in habitat structure, microclimate, and resource availability, which leads to alterations in species composition, abundance, and interactions within ecosystems (Didham, Tylianakis, Gemmell, Rand, & Ewers, 2007; Fitzherbert et al., 2008; Franco et al., 2006; Gaston, Blackburn, & Goldewijk, 2003; Tilman, 1994; Turner & Foster, 2009; Tylianakis et al., 2007, 2008). Changes in species occurrence within a habitat can result in profound impacts to predator–prey, host–parasite, or other species interactions (Aerts, 1999; Kneitel & Chase, 2004; Tilman, 1994; Wright, 2002; Zobel, 1992). Understanding and conserving species interactions is critical to the maintenance of species richness and ecosystem functioning as habitats are modified and is of paramount importance in agricultural ecosystems, as humans rely on many of these interactions for services such as pollination and pest control (Costanza et al., 1997, 2014).

Altered host–parasite relationships can cause additional changes to already modified ecosystems, especially if hosts or parasites are functionally important species (Kutz, Hoberg, Polley, & Jenkins, 2005; Nazzi et al., 2012; Sammataro, Gerson, & Needham, 2000). In agroecosystems, many important pollinators, pests, and pest control agents are parasites or hosts (Nazzi et al., 2012; Sammataro et al., 2000; Sheffield, Pindar, Packer, & Kevan, 2013; Tscharntke et al., 2007). Species losses or changes to resource availability alter host–parasite interactions through changes to host density, host fitness, prey availability, and the level of intra‐ or inter‐specific competition (Barber & Martin, 1997; Berndt, Wratten, & Scarratt, 2006; Hahn & Hatfield, 1995; Irvin et al., 2006; Kruess, 2003; Rusch, Valantin‐Morison, Sarthou, & Roger‐Estrade, 2011; Tilman, 1994; Tylianakis et al., 2007; Wilkinson & Feener, 2007; Wolinska & King, 2009). Increased habitat complexity can increase parasitism rates by providing additional resources for parasites, such as increased host density or food resources (Berndt et al., 2006; Irvin et al., 2006; Kruess, 2003; Rusch et al., 2011; Tylianakis, Didham, & Wratten, 2004), but can also decrease parasitism rates by increasing the host's ability to defend against parasites (Denno, Finke, & Langellotto, 2005; Gols et al., 2005; Wilkinson & Feener, 2007).

Cleptoparasites such as *Argyrodes spp*. (Araneae: Theridiidae) spiders may be particularly sensitive to habitat change due to their direct reliance on host spider success (Sheffield et al., 2013). *Argyrodes* are obligate cleptoparasitic spiders that depend solely on food resources caught by their hosts and living space provided by their hosts (Agnarsson, 2003; Cangialosi, 1997; Vollrath, 1987b). Yet, *Argyrodes* can also have profound negative effects on host fitness (Elgar, 1989; McCrate & Uetz, 2010; Rittschof & Ruggles, 2010; Rypstra, 1981; Tanaka, 1984). *Argyrodes* reach densities of up to 40 individuals per square meter of their host's web, and even at much lower densities, can consume enough prey to significantly impact host growth, web tenure, web damage, and host mortality (Agnarsson, 2003, 2011; Grostal & Walter, 1997; Koh & Li, 2002; Miyashita, 2001; Rypstra, 1981). *Argyrodes* may also indirectly reduce host reproductive success: Male *Nephila* spiders, which are substantially smaller than females and co‐occupy female webs, also act as cleptoparasites, thereby directly competing with resident *Argyrodes* for access to food (Christenson, Brown, Wenzl, Hill, & Goist, 1985). Because of the sensitive balance of competition with and dependence on hosts, any change in the interactions between cleptoparasites and their hosts could be an early indicator of changing trophic interactions within modified ecosystems (Sheffield et al., 2013; Tylianakis et al., 2007, 2008). In addition, owing to their role as generalist predators, any changes in the trophic interactions of spiders have the potential to impact pest control in agricultural systems (Cardinale, Harvey, Gross, & Ives, 2003; Denno et al., 2005; Jonsson, Wratten, Landis, & Gurr, 2008). While *Argyrodes,* because they consume insects already caught in host webs, are not likely to be critical pest control agents, their impacts on *Nephila* host health or behavior have the potential to alter host pest control potential. Although little is yet known about the role of spiders in oil palm specifically, they can be important pest control agents in other tropical (Hlivko & Rypstra, 2003; Kobayashi, 1975; Settle et al., 1996; Sigsgaard, 2000) and tree (Mansour, Rosen, & Shulov, 1980; Mathews, Bottrell, & Brown, 2004) crops.

*Argyrodes* abundance is known to be positively correlated with web size, host body size, host density, and prey availability (Agnarsson, 2003, 2011; Cangialosi, 1990, 1991; Elgar, 1989; Grostal & Walter, 1997; Hénaut, Delme, Legal, & Williams, 2005; Koh & Li, 2002), but the overarching effects of habitat structure on density and abundance are less well understood (Agnarsson, 2011; Cangialosi, 1997; Miyashita, 2002; Rittschof & Ruggles, 2010). Here, we present the first study on the effects of habitat complexity in an oil palm agroecosystem on host–parasite interactions between a spider and a cleptoparasite. We examine the impact of oil palm understory vegetation complexity as well as host characteristics (host web size and male *Nephila* presence) on *Argyrodes miniaceus* (Doleschall, 1857) cleptoparasites in the webs of female *Nephila spp*. hosts (Koh & Li, 2002; Miyashita, 2002; Rypstra 1981). We make the following hypotheses about the effects of these environmental factors on *A. miniaceus* density and abundance:

1. Greater vegetation complexity allows for greater cleptoparasite density and abundance.
2. Cleptoparasite density is constant or greater in larger webs.
3. As male *Nephila* also act as cleptoparasites in female webs, and so benefit from similar environmental conditions to *Argyrodes,* their presence is positively associated with *Argyrodes* density.

By quantifying the effect of understory complexity on a host–parasite relationship, this study will yield novel insights into the effect of habitat structure and management on food web complexity in a tropical agricultural landscape.

## 2 MATERIALS AND METHODS

### 2.1 Study site

This study was conducted as part of the Biodiversity and Ecosystem Function in Tropical Agriculture (BEFTA) Project, located in Riau Province, Sumatra, Indonesia (Foster et al., 2014). The BEFTA Project is a large‐scale, long‐term ecological experiment testing the effects of understory vegetation management on oil palm biodiversity, ecosystem functions, and yield. It is being conducted in oil palm estates owned and managed by PT Ivo Mas Tunggal, a subsidiary company of Golden Agri Resources (GAR), with technical advice from Sinar Mas Agro Resources and Technology Research Institute (SMARTRI, the research and development center of GAR). The area surrounding the estates consists primarily of oil palm plantations, with a small coverage of other crops.

### 2.2 Experimental treatments

Eighteen study plots were established in October 2012. Oil palm trees on all plots were planted between 1987 and 1993, and so were mature at the time of the study. Plots are 150 m by 150 m and are located on flat ground between 10 and 30 m above sea level and without adjacent human habitation. The plots are arranged in triplets, with one plot in each triplet randomly assigned a different understory vegetation management treatment. Treatments were implemented in February 2014, and involved the following management:

1. Standard understory complexity: standard company practice, consisting of intermediate herbicide use, and understory vegetation removal.
2. Reduced understory complexity: intensive herbicide use and removal of understory vegetation.
3. Enhanced understory complexity: no herbicide treatment and minimal understory vegetation removal.

Herbicides used in the initial establishment of the plots included Glyphosate (Rollup 480 SL), Paraquat Dichloride (Rolixone 276 SL), metsulfuron‐methyl (Erkafuron 20 WG) and Fluroxypyr (Starane 290 EC). These were sprayed exclusively on the ground vegetation and are unlikely to have directly affected *Nephila* webs. Although there is little evidence regarding the direct toxic effects of properly applied herbicide on invertebrates (Marshall, Brown, Boatman, Lutman, & Squire, 2001), at least one study indicates little to no direct effect of herbicide application on leaf‐litter invertebrate communities (Lindsay & French, 2004).

### 2.3 Sampling protocol

To measure cleptoparasite density, we walked along every row of oil palm trees in each 150 m x 150 m plot in March 2016, two years after the experimental treatments had been implemented. Tree rows were planted approximately 8 m apart, with each tree in a row also approximately 8 m apart. We noted every adult *Nephila spp*. web within the plot that was less than 3 m above the observer's head and was over 10 cm in both length and width. Species of *Nephila* present in the study plots included *N. pilipes* (Fabricius, 1793) and *N. kuhlii* (Doleschal, 1859). *Argyrodes miniaceus* was the only species of *Argyrodes* present on *Nephila spp*. webs in the study plots. Webs consist of a large central orb surrounded by varying amounts of barrier webbing. To obtain a rough estimate of the size of the web, we measured the length and width of the central orb of every web. We did not measure the size of barrier webbing, and we did not measure distances between webs, although we observed no interconnected webs. We counted all *A. miniaceus* cleptoparasites that were within the central orb and on the surrounding barrier webbing, including any web attachment threads. We also noted whether or not any male *Nephila* were present in the web of the larger female host.

### 2.4 Analyses

All analyses were conducted using R version 3.3.0 (R‐Core‐Team 2016). We calculated cleptoparasite density per web by dividing the number of cleptoparasites by the web area in square meters (calculated as orb length × orb width). To determine what factors best predict cleptoparasite density, we created a linear mixed effects model with square root transformed cleptoparasite density data as the response variable, using the R package “lme4” (Bates, Maechler, Bolker, & Walker, 2015). We tested treatment, host web size (m^2^), and the presence of a male in the host web as potential explanatory variables and included triplet as a random effect.

We used an automatic drop‐in‐deviance test to select the best‐fit model. The automatic drop‐in‐deviance test used a series of likelihood ratio tests to determine which variables most improved the model's goodness of fit. We chose the variable with the lowest reported Akaike's Information Criterion (AIC) to add to the model and repeated the test, adding variables one at a time until no more factors were significant at the α = 0.05 level (Burnham & Anderson, 1998). We then used additional likelihood ratio tests to determine whether including interactions between any of the selected explanatory variables increased the model's goodness‐of‐fit. The final model was the model with the lowest AIC value.

To examine differences in mean cleptoparasite density per web and abundance per plot among treatments, we conducted ANOVA tests on square‐root transformed density data and untransformed abundance data, with triplet as a random effect. We square root transformed the density data to avoid violating assumptions of normality of residuals and homoscedasticity. Because the effect of a treatment can be underestimated by *p*‐values for small sample sizes (Gelman & Stern, 2006; Ioannidis, 2005), we considered results marginally significant for *p*‐values below α = 0.1 for ANOVA tests on per‐plot abundance (where *n* = 6 plots). After any significant or marginally significant results, additional ANOVAs were used to test pairwise differences in *A. miniaceus* density and abundance between treatments.

## 3 RESULTS

**Figure 1:**
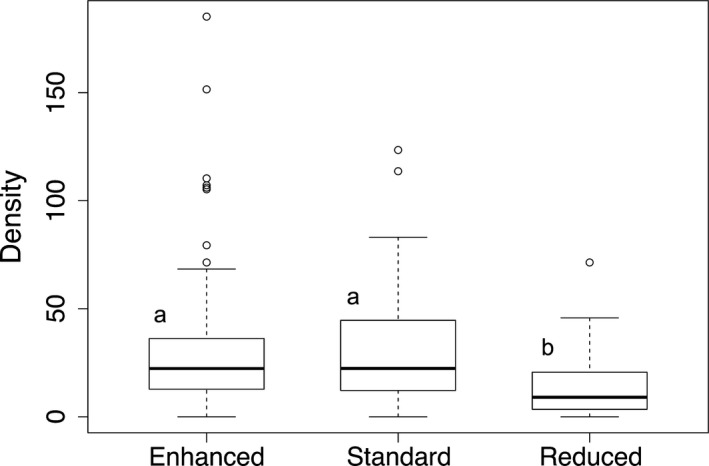
Boxplots depicting medians and interquartile ranges of *Argyrodes miniaceus* cleptoparasite density per square meter of *Nephila spp*. web in BEFTA Project plots of enhanced complexity (*n* = 89 webs), standard complexity (*n* = 96 webs), and reduced complexity (*n* = 28 webs) understory management treatments. Letters (a or b) indicate significant differences in means: a different letter indicates a difference from the others

**Figure 2:**
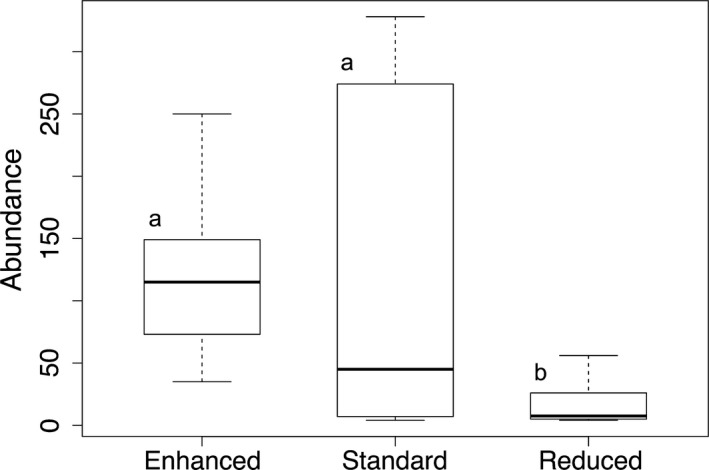
Boxplots depicting medians and interquartile ranges of *Argyrodes miniaceus* cleptoparasite abundance per plot in BEFTA Project plots of enhanced complexity (*n* = 6 plots), standard complexity (*n* = 6 plots), and reduced complexity (*n* = 6 plots) understory management treatments. Letters (a or b) indicate significant or marginally significant differences in means: a different letter indicates a difference from the others

We counted a total of 737 *A. miniaceus* individuals in 89 *Nephila* host webs in enhanced complexity plots, 703 individuals in 96 webs in standard complexity plots, and 106 individuals in 28 webs in reduced complexity plots. Six webs in enhanced complexity plots, six webs in standard complexity plots, and five webs in reduced complexity plots did not contain cleptoparasites. Webs that did not contain cleptoparasites ranged in size from 400 cm^2^ to over 2000 cm^2^. The maximum number of cleptoparasites found in one web was 34 in enhanced complexity plots, 27 in standard complexity plots, and 13 in reduced complexity plots. Mean cleptoparasite density was 31.4 ± 3.31 *A. miniaceus* per square meter of *Nephila* web in enhanced complexity plots, 29.3 ± 2.46 per square meter of web in standard complexity plots, and 14.4 ± 3.02 per square meter of web in reduced complexity plots (mean ± *SE*; Figure 1). Mean abundance of cleptoparasites per plot in the enhanced complexity treatment was 122.8 ± 31.21, in the standard complexity treatment was 117.2 ± 59.47, and in the reduced complexity treatment was 17.7 ± 8.38 (mean ± *SE*; Figure 2).

Treatment, web size, and the presence of a male *Nephila* were all significant predictors of cleptoparasite density and were thus all included in the final model (Table 1). No interactions between variables were significant. Model parameter coefficients indicate that, on average, larger webs have lower cleptoparasite densities, and male presence is associated with higher cleptoparasite densities (Table 1).

**Table 1:** Parameter coefficients (±*SE*) and random effect with variance (±*SD*) of the best‐fit linear model predicting *Argyrodes miniaceus* cleptoparasite density per square meter of *Nephila spp*. web in BEFTA Project plots

Variable	Coefficient (±*SE*)
Web size (m^2^)	−2.853 (±0.663)
Male *Nephila* presence	1.047 (±0.465)
Reduced complexity treatment	−1.979 (±0.503)
Standard complexity treatment	−0.346 (±0.351)
Enhanced complexity treatment^a^	0 (± 0)^a^

Random Effect	Variance (±*SD*)
Triplet	0.139 (±0.373)

a Enhanced complexity treatment was used as the reference category during model construction, so was assigned a coefficient of zero.

Mean *A. miniaceus* density per square meter of *Nephila spp*. host web differed across treatments (*F = *8.13, *df* = 2, *p < *.001) and was significantly lower in reduced complexity plots than in both enhanced complexity plots (*F *=* *18.75, *df* = 1, *p *<* *.001) and standard complexity plots (*F *=* *11.79, *df* = 1, *p *<* *.001; Figure 1). Density in enhanced and standard complexity plots did not significantly differ (*F *=* *2.20, *df* = 1, *p *=* *.14; Figure 1). Mean *A. miniaceus* abundance per plot was marginally significantly different across treatments (*F *=* *3.75, *df* = 2, *p *=* *.061; Figure 2). Abundance in enhanced complexity plots was significantly greater than abundance in reduced complexity plots (*F *=* *15.29, *df* = 1, *p *=* *.011; Figure 2). Abundance in standard complexity and reduced complexity plots did not differ (*F *=* *3.07, *df* = 1, *p *=* *.14), nor did abundance differ between standard and enhanced complexity plots (*F *=* *0.019, *df* = 1, *p *=* *.90; Figure 2).

## 4 DISCUSSION

Habitat complexity in agroecosystems can have profound effects on species interactions, with potential impacts on the provision of ecosystem services (Finke & Denno, 2002; Langellotto, 2002; Martin, Reineking, Seo, & Steffan‐Dewenter, 2015). This study investigated factors, including habitat complexity, that influence *Argyrodes miniaceus* cleptoparasite occurrence within *Nephila spp*. host webs in oil palm plantations. Understory vegetation complexity, host web size, and male *Nephila* presence were all significant predictors of *A. miniaceus* density in *Nephila spp*. host webs. Greater cleptoparasite density was associated with enhanced and standard levels of understory complexity, smaller webs, and the presence of a male *Nephila spp*. in the web. In addition, total cleptoparasite abundance differed across understory complexity treatments, with significantly fewer cleptoparasites in reduced understory complexity plots.

### 4.1 Cleptoparasite density and vegetation complexity

Our results suggest that greater habitat complexity in oil palm plantations supports higher populations of cleptoparasitic spiders, both per host web and in terms of total abundance. This finding adds to a body of literature demonstrating that vegetation complexity and diversity support complex food webs, although these studies were primarily conducted in temperate ecosystems (Macfadyen, Gibson, Symondson, & Memmott, 2011; Memmott et al., 2007; Pocock, Evans, & Memmott, 2012). For example, a study by Goulson, Hughes, Derwent, and Stout (2002) found that an increase in floral resources in suburban and agricultural habitats in the United Kingdom increased both the abundance of native bumblebees and the abundance of their specialist parasites. Ebeling, Klein, Weisser, and Tscharntke (2012) also report that greater plant diversity in German grasslands increases not only the diversity of host bee and wasp species, but the diversity of their parasitoids as well. Our study indicates that a similar relationship exists in tropical agroecosystems.

An increased abundance of *Nephila spp*. host webs in enhanced and standard complexity plots compared to reduced complexity plots (89 *cf*. 96, *cf*. 28) is one probable cause of the observed increase in abundance of *A. miniaceus*. Enhanced vegetation complexity may provide greater prey availability, which could account for the greater abundance of *Nephila* host webs as well as the higher density per web and total abundance of *Argyrodes* within enhanced and standard complexity plots. Higher abundances of arthropods, including carabid and rove beetles, aphids, mites, Lepidopterans, and several types of Hemipterans, have been found in habitats with greater vegetation complexity and diversity (Andow, 1991; Chaplin‐Kramer, O'Rourke, Blitzer, & Kremen, 2011; Hansen, 2000; Landis, Wratten, & Gurr, 2000; Langellotto & Denno, 2004; Weibull, Östman, & Granqvist, 2003). *Argyrodes* populations are limited by competition for food resources—both with the host and with other cleptoparasites (Miyashita, 2001; Whitehouse, 1997)—and so an increased abundance of prey would allow each *Nephila* web to support a greater density of *Argyrodes* cleptoparasites (Cangialosi, 1991; Miyashita, 2001; Whitehouse, 1988, 1997).

Distance between host webs may also play a role in cleptoparasite density. Isolated habitat patches are less likely to be inhabited by any given species (Prugh, Hodges, Sinclair, & Brashares, 2008; Watling & Donnelly, 2006) and are expected to exhibit high extinction and low immigration rates (Brown & Kodric‐Brown, 1977; Fahrig & Merriam, 1985; Hanski, 1999). Although we did not directly measure distances between host webs, the observed lower abundance and therefore density of *Nephila* host webs in reduced complexity plots suggests webs in these plots are more isolated than in the densely populated enhanced complexity plots. If we consider host webs as habitat patches, the isolation of host webs in reduced complexity plots may be an additional contributing factor to lower cleptoparasite density. However, previous research has indicated that host web isolation does not correlate with cleptoparasite abundance (Agnarsson, 2011). Future research might more closely examine the relationship between host web inter‐distance and cleptoparasite density.

### 4.2 Web size, male presence, and cleptoparasite density

*Argyrodes miniaceus* density was also correlated with host web size: Density was greater in smaller webs. Previous research suggests that cleptoparasite density should remain constant across host webs, due to the strong linear relationship between web size and cleptoparasite abundance (Agnarsson, 2011). In several studies, removal or addition of *Argyrodes spp*. individuals to host webs resulted in nearly immediate immigration to or emigration from the web, keeping density constant (Miyashita, 2001, 2002; Rypstra, 1985). *Argyrodes* often behave aggressively toward each other, with population density limited by competition for food and space (Miyashita, 2001, 2002). The observed disparity in cleptoparasite density among host webs of different sizes is therefore surprising. We found no significant differences in web size across treatments (D.M. Spear unpublished data), and so the observed trend cannot be explained by disparities in average *Nephila* web size across our treatments. Female *Nephila* host body size may be a contributing factor to the observed differences in density. Large webs are typically occupied by larger or older hosts (Eberhard, 1972; Grostal & Walter, 1999; Kuntner, Gregorič, & Li, 2010; Moore, 1977; Witt, Reed, & Peakall, 2012), which can behave more aggressively toward cleptoparasites than younger “naïve” females, predating upon or chasing occupants from webs (Cangialosi, 1991; Vollrath, 1979, 1987b; Whitehouse, 1988, 1997). It is possible that these older hosts may more effectively limit the density of *Argyrodes* within their webs. Further research is necessary to determine the potential interactive effects of host web size and habitat complexity on cleptoparasite density.

The correlation between male *Nephila* presence and *Argyrodes* density may also reflect differences in food resources among treatments. Males frequently act as cleptoparasites in females' webs, and so benefit from similar factors to cleptoparasites, such as prey abundance (Christenson et al., 1985; Elgar et al., 2003; Vollrath, 1987b). Webs that are preferable to males may be located in areas of high resource abundance, and so may be equally suitable for high densities of cleptoparasites. Grostal and Walter (1999), who similarly found a positive correlation between presence of *Nephila plumipes* host males and abundance of *Argyrodes antipodianus* cleptoparasites, also propose that males may distract the female host, thus decreasing the risk of predation by *Nephila* females for *Argyrodes* inhabitants and making male‐occupied webs safer. An increased cleptoparasite load therefore may constitute an additional cost of reproduction for *Nephila* females.

### 4.3 Impacts of changing cleptoparasite occurrence

High *Argyrodes* density could have significant impacts on hosts, including decreased web tenure, increased web damage, decreased prey consumption rate, decreased growth rate, and increased mortality (Agnarsson, 2003, 2011; Grostal & Walter, 1997; Koh & Li, 2002; Miyashita, 2001; Rypstra, 1981). All of these effects have the potential to decrease the total rate of prey capture and the rate of reproduction of host spider populations (Chmiel, Herberstein, & Elgar, 2000; Elgar, 1989; Miyashita, 1986; Rypstra, 1981; Vollrath, 1987a). Such a decrease in host fitness could diminish the ecosystem services these spiders provide by reducing capture of pest or other arthropod species (Rusch, Birkhofer, Bommarco, Smith, & Ekbom, 2015; Symondson, Sunderland, & Greenstone, 2002; Tscharntke et al., 2007).

However, high occurrence of both *Nephila spp*. and cleptoparasites in enhanced and standard complexity plots suggests that any detrimental effects of an increased cleptoparasite load are not severe enough to significantly limit *Nephila* population levels. Resource availability in enhanced complexity plots must therefore be great enough to support both high cleptoparasite abundance and high *Nephila* density, providing evidence that higher vegetation complexity increases the ability of oil palm ecosystems to support both more predators and more complex trophic interactions. The lower densities of cleptoparasites per web area in reduced complexity plots suggest that fewer resources were available to support either the hosts or the parasites. Because cleptoparasites rely on taking any remaining food once their host's energy requirements are met, cleptoparasites are likely to be more sensitive to habitat change than their hosts (Sheffield et al., 2013). Thus, changes to cleptoparasite abundance could be a first indicator of changing trophic interactions across modified ecosystems (Sheffield et al., 2013; Tylianakis et al., 2007, 2008).

## 5 CONCLUSIONS

While increased vegetation complexity and diversity provides beneficial resources to spiders in agricultural systems (Diehl, Mader, Wolters, & Birkhofer, 2013; Rypstra, Carter, Balfour, & Marshall, 1999; Schmidt, Roschewitz, Thies, & Tscharntke, 2005), this study shows that vegetation complexity also has the potential to increase cleptoparasite loads. The most likely reason for this is that maintaining understory vegetation in oil palm plantations provides additional resources that support a greater abundance and density of *Nephila* hosts, and also a greater number of cleptoparasites per web. This study demonstrates the potential of within‐plantation management to increase the complexity of tropical food webs, increasing the abundance of both predators and the parasites they support, with potential impacts on the ecosystem services that predators provide.

## CONFLICT OF INTEREST

None declared.

## AUTHORS' CONTRIBUTIONS

DMS helped design the study, conducted the fieldwork, analyzed data, and drafted the manuscript; ECT and WAF coordinated and helped with all aspects of the study; SHL helped design the study, draft the manuscript, and analyzed the data; ADA helped design the study and conduct fieldwork; MN, JPC, JLS, ADA, and SP all helped design the study, and helped design, coordinate, and maintain BEFTA Project plots and treatments. All authors contributed critically to the drafts and gave final approval for publication.

## DATA ACCESSIBILITY

The data used in this study will be archived in the Dryad Digital Repository.
